# Drug-safety reporting in Polish nursing practice—Cross sectional surveys

**DOI:** 10.1371/journal.pone.0241377

**Published:** 2020-10-27

**Authors:** Agnieszka Zimmermann, Agata Flis, Aleksandra Gaworska–Krzemińska, Marsha N. Cohen

**Affiliations:** 1 Department of Medical and Pharmacy Law, Medical University of Gdańsk, Gdańsk, Poland; 2 Department of Nursing Management, Medical University of Gdańsk, Gdańsk, Poland; 3 University of California Hastings College of the Law, San Francisco, California, United States of America; University of Oxford, UNITED KINGDOM

## Abstract

**Introduction:**

Nurses play a significant role in ensuring the safety and quality of drugs. Our aim was to assess significant factors in nurses’ participation in ensuring pharmacotherapy safety by reporting adverse drug reactions (ADR) and detecting substandard drugs (SD).

**Materials and methods:**

The study was a cross-sectional, comparative survey, using original questionnaires. Survey questions were grouped to probe the opinions, attitudes and practices of nurses reporting ADRs and SDs. Data were obtained from nurses working in teaching hospitals in Poland (group A) and, for comparison, in the USA (group B). 1200 questionnaires were distributed in Poland (return rate: 55.7%) and 200 questionnaires in the USA (return rate: 73%). Both groups were surveyed during the same period. There were no exclusion criteria. The questionnaires were self-administered. Distribution and collection were anonymous. Participation was voluntary. The Spearman correlation test was used. Both groups’ responses were cross-tabulated and compared using Fisher’s Exact Test for Count Data.

**Results:**

The study group comprised 669 Polish and 146 American professionally active nurses working in general care and surgical departments. Age range: 18 to 72 years. Median job seniority: 18.3 years (group A) and 20.6 years (group B). Education levels varied. ADR reporting conditions in Poland are unfavorable: shortage of time—83.9% vs 22.6% in the US (p = 0.01); no incentive—58.2% vs 6.1% in the US (p = 0.01); and no equipment—44.7% vs 2.8% in the US (p < 0.01). Both Polish and American nurses indicate they rarely report SDs, with rates of 0.4% and 11% (p < 0.0001) respectively, during the study period.

**Conclusions:**

Nurses in Poland are insufficiently prepared to ensure drug safety conscientiously and responsibly. Training is required for Polish nurses. Nurses’ employers need to improve conditions to enable reporting of ADRs and SDs.

## 1. Introduction

Apart from the efficacy of the therapy itself, ensuring patient safety is the primary aim in patient care. The International Council of Nurses policy on nursing care indicates that patient safety is a priority, including the elimination, prevention, and reduction of unintended harm associated with pharmacotherapy [1, 2]. Nurses ought to be active in pharmacovigilance (PhV) and report adverse drug reactions (ADRs), as the spontaneous reporting of ADRs is a foundation of post-marketing drug safety surveillance [3, 4]. Widely utilized worldwide, spontaneous reporting of ADRs is one of the cheapest methods of monitoring the safety of medicines; and therefore improving strategies for spontaneous ADR reporting is a key to reducing the risks, and increasing the benefits, of drug therapies. Despite its importance for patient safety, only approximately 10% of all ADRs cases are reported [5].

Further, although physicians, pharmacists, nurses and midwives in Poland are obligated to report ADRs, there is a low rate of ADR reporting which is at odds with the high levels of drug consumption [6]. This is confirmed by the Office for Registration of Medicinal Products, Medical Devices and Biocidal Products (ORMP) annual reports in Poland: in 2016 there were 19,656 reports, of which, only 2,433 came from health care providers; in 2017 there were 19,996 reports and only 2,857 of these were from health care providers; and in 2018: 21,425 reporst with only 3,270 from health care providers. These data do not indicate how many nurses are involved in the reporting, and it is our understanding that most reports originate with physicians [7], though it is known that the rate of ADRs reporting by nurses in Poland is unsatisfactorily low. Increased involvement of nurses in reporting adverse reactions ought to contribute to patient safety and reductions in the costs of the treatment of ADRs complications. Nurses’ close contact with patients gives them the privilege of identifying adverse reactions and providing valuable information on drug safety [8, 9].

The literature shows that ADRs reporting improves with education and training, motivational strategies, and enhanced access to reporting tools, as these factors address the main causes of under-reporting ADRs [10]. However, in Poland, the reasons for under-reporting among nurses has not been unconfirmed as there are only a few studies have investigated ADRs reporting, and none have been undertaken in nursing settings. This was one reason we conducted our study.

In addition, nurses have a significant role in the detection of substandard drugs (SDs) prior to them being administered. In Poland, nurses are authorized to report problems with the quality of any medicine to the pharmaceutical inspectorate. Each year in Poland, between 80 and 150 SDs are recalled from the market, and while these decisions and the reasons are made public, there is no data on the source of the originating SDs report [11], and there have been no studies of nurses’ involvement in SDs detection in Poland. This was the other reason we conducted our study.

Our objective was to analyze the factors that play a significant role in nurses’ active participation in ensuring safety pharmacotherapy by their reporting ADRs and detecting SDs in clinical practice.

## 2. Materials and methods

### 2.1. Research design, participants and study setting

Our study utilized a cross-sectional design. Data were obtained from two sources including nurse surveys distributed among Polish nurses (group A) and, for comparison, American nurses (group B). Both groups of respondents were nursing staff of urban tertiary care teaching hospitals. It was assumed that nurses working in the United States systematically report adverse drug reactions. The researchers calculated the required sample size for the questionnaire on the following bases. At the time of the project, there were 221,172 nurses employed in Poland. It was determined that a minimum sample size of 384 completed questionnaires was needed to achieve a confidence level of 95% and that the real value is within ±5% of the confidence interval. In comparison, a minimum sample size of 100 was established for group B.

The study tools were created by the authors of the study based on the available literature. When designing the research tool we used a content validity measure: during the survey design, 6 scientists from the Sociology Department were asked to rate the survey questions and categorize them as either “essential”, “useful”, or “not necessary”. A Content Validity Ratio was calculated for each question, and the results ranged from 0.6 to 0.9. Principal component factor analysis was used to uncover the underlying dimensions of the Likert scale. This analysis provided the basis for researchers using the 5 response levels. The questionnaires included 11 questions grouped into sets to probe for the opinions and attitudes of the nurses, and questions to analyze nurses’ practice of reporting ADRs and detecting SDs. Survey items were divided into 4 major domains plus a section for socio-demographic and professional data. In the first section, “Opinions”, two questions probing for nurses’ opinions were provided, each with five response levels corresponding to the Likert scale, ranging from “1-Strongly disagree” to “5-Strongly agree”. These questions sought subjective assessments of:

- the safety of the medicinal products being used, and
- the nurse’s professional preparation to report ADRs and SDs.

In the second section, three questions were asked, each with five response levels corresponding to the Likert scale, analyzing workplace characteristics:

- time that could be spent on reporting ADRs and SDs,
- the availability of equipment and forms required to report ADRs and SDs, and
- the employer’s system for motivating nurses to report ADRs and SDs.

The third section consisted of 3 questions aimed at measuring nurses’ attitudes towards reporting ADRs and SDs, each with five response levels corresponding to the Likert scale. The questions were about:

- the duty to report ADRs and SDs,
- the impact that reporting single cases of ADRs and SDs has on the safety of pharmacotherapy, and
- additional risks associated with ADR and SD reporting, e.g., being held responsible for a potential error.

In the fourth section, aimed at being able to analyze nurses’ reporting practice, 3 questions sought answers to:

- whether the respondents had reported any ADRs in the previous three years,
- why the respondents had not reported ADRs despite observing them (possible answers: “if they are described in the package leaflet”, “if they are not bothersome for the patient”, if “I am sure that they are not associated with the given drug”), and
- whether the respondents had detected any SDs in the previous three years; and if so, with what frequency: once a year; twice a year; or once, twice or three times during the entire period of employment.

Validation of the questionnaire first addressed the time that would be needed to complete it; and then assessing the clarity and intelligibility of its contents. Doubts or suggestions by validation respondents were helpful in clarifying the draft questions in the questionnaire. The internal consistency of those questions using the Likert scale was examined using the Alpha-Cronbach coefficient; and a significant, high correlation was found between the results obtained for each question and the total number of points. The calculated ratio was high, at 0.85, which indicated there was a high degree of internal consistency in the questionnaire. Then the revised questionnaire was validated for a second time with a group of 20 professionally active nurses, all members of the Gdańsk branch of the Polish Nursing Society, who had been invited to participate by e-mail. All their comments were taken into consideration. Then we conducted a pilot study of the Polish language questionnaire to identify any potential problems or deficiencies in the research instruments prior to implementation with the full study group. For our study, the survey was translated into English and, to check for accuracy, back to Polish by two different certified translators (forward–backward translation). To assess the translational and conceptual equivalence of the survey questions, the translated questionnaire was shown to 4 experienced, graduated, English-only speaking, US nurses with recent clinical experience to ensure that the clinical meaning was appropriate. They were asked to evaluate the relevance of the questions to nursing practice. The standard four-point content validity indexing rating scale was used to evaluate each of the questions from the translated questionnaire (1 = Not relevant, 2 = Somewhat relevant, 3 = Very relevant, 4 = Highly relevant). The entire process was managed online through email. The survey received mostly ratings of 3 and 4 for each item in the questionnaire. In the validation and pilot studies of both the Polish and English versions of the questionnaire we maintained an identical format and layout. We conducted a pilot study of the English language version of the questionnaire with 10 nurses from the American Society of Registered Nurses (San Francisco) by email.

Finally, the survey was conducted among nurses working in all five teaching hospitals in northern Poland (at Białystok, Bydgoszcz, Gdańsk, Olsztyn, and Szczecin) that work with the two corresponding Regional Centres of Monitoring the Adverse Drugs Reactions (in Gdańsk and in Szczecin)–group A. In each of the five cities, there is a pharmaceutical inspectorate responsible for SD detection. With the group B survey, nurses employed at Vidant Medical Center, Greenville, North Carolina, USA, and that cooperates with the East Carolina University College of Nursing, were invited to take part in the trial using the English questionnaire.

### 2.2. Data collection procedures

In the study participation was voluntary. Invitations to take a part in a survey were sent to the hospitals identified in the previous section, and once hospital managers granted their consent, questionnaires in paper form were sent to the hospitals for distribution to nurses with a „Survey Participation Consent Form” which was used to gain participants’ written informed consent. In total, 1200 questionnaires were distributed in Poland, with a return rate of 55.7%. Once they were completed, the head nurse returned the questionnaires via post. In total, 200 paper questionnaires were distributed to the American nurses and they were subsequently collected at the hospital in a special locked box. The rate of completed and returned questionnaires in the USA was 73%. The survey was administered during the same period for both groups. The research was conducted from July to December 2018. There were no exclusion criteria. The participants in our study were guaranteed complete anonymity.

### 2.3. Ethical considerations

The project was approved by the Independent Bioethics Committee for Scientific Research at Medical University of Gdańsk as not raising any ethical issues (NKBBN/45/2015), including an assessment that the project constituted “non-invasive research”. The study was carried out with the consent of the managing directors of the participating facilities. All our study subjects gave their written informed consent prior to participation. The study was conducted in accordance with the requirements of the data protection legislation in Poland and the United States of America. Participants received both written and oral information about the study. All the questionnaires were collected in locked boxes and subsequently stored in a locked office.

### 2.4. Data processing and analysis

Questions with no answers (non-response item) were excluded from the analysis. Data collected was digitized manually and a unique number was generated for each questionnaire by a person responsible for entering the data into an Excel database. The accuracy of coding and data entry was checked by a second person from the research team. All statistical calculations were carried out using data analysis software system STATISTICA version 12.0 (StatSoft. Inc, 2014, www.statsoft.com) and an Excel spreadsheet. Pearson’s and/or Spearman’s correlation coefficients were used to verify the existence, strength, and direction of relationships between variables: age, job seniority, educational level, and place of work. The Spearman correlation test was used as appropriate for measurements taken from ordinal scales. Pearson correlation was used to assess agreement between two continuous measurements (in the Polish and American groups). Spearman correlation was chosen as appropriate for small samples. Both groups’ responses were cross-tabulated and compared using Fisher’s Exact Test for Count Data. The level of significance in all calculations was assumed to be p < 0.05.

## 3. Results

Group A included 669 nurses practicing in Poland. The total number of participant nurses exceeded the minimum required sample size. There were 656 women (98%) and 13 men (2%) involved in the questionnaire study, which reflects the actual gender ratio of working nurses in Poland. In group B, 146 nurses participated in the study: 144 women (98.6%) and 2 men (1.4%). The declared ages ranged from 22 to 62 years in the Polish group (women used to retire at 60 years old in Poland) and from 18 to 72 years in group B. These were professionally active nurses working in general care (64% vs 62%, for groups A and B respectively) and surgical departments (36% vs 38%). The average length of professional service was 18.3 years in the Polish group and 20.6 in the American group. The highest education levels of respondents in group A: secondary education (35.6%), bachelor’s degree (38.8%) and master’s degree (25.6%); and in group B: licensed practical nurses (43.2%), registered nurses (43.1%), advanced registered nurses practitioners (13.7%). As many as 89.9% vs 80.2% (in group A and B respectively) of the respondents did not participate in any training concerning reporting ADRs during the three years prior to their participation in our study.

The comparison of nurses’ opinions on the safety of medicinal products revealed that 71% of Polish nurses and 50.7% of American nurses respectively considered drugs to be “rather” safe; and 8.2% Polish nurses compared with 38.4% American nurses considered drugs to be “definitely” safe. Respondents who selected the answer “I do not know”: 17.1% Polish versus 8.8% American nurses. It was proven that American nurses rated the safety of medicinal products being used as significantly higher than nurses working in Poland (p < 0.001). In the Fischer test, p-value < 0.001. These data are presented in Table 1.

**Table 1:** Responses of the Polish (group A) and American (group B) nurses to the question: “How do you rate the safety of medicinal products being used?”.

**Responses**	**Group A (n = 669)**	**Group B (n = 146)**	**p-value**
Somewhat dangerous	25 (3.7%)	3 (2.1%)	**p < 0.001**
No opinion/Don’t know	114 (17.1%)	13 (8.8%)	
Somewhat safe	475 (71.0%)	74 (50.7%)	
Definitely safe	55 (8.2%)	56 (38.4%)	
**Response characteristics**	**Statistical values**		
Mean (SD)	3.8 (0.6)	4.3 (0.7)	
Range	2.0–5.0	2.0–5.0	
Median	4.0	4.0	

* p value is for the comparison of proportions.

43.2% of Polish nurses compared with 4.8% of American nurses responded “strongly disagree” or “disagree” to the question about being adequately prepared to independently report ADRs and SDs. Those who responded with “agree” and “strongly agree” were 25.9% of Polish nurses and 92.5% of American nurses (p-value = 0.01). Comparing both groups using Fisher’s exact test showed a p-value < 2.2•10^−16^. The comparison of responses is shown in Table 2.

**Table 2:** Responses of the Polish (group A) and the American (group B) nurses to the question: “Do you think that nurses are adequately prepared to independently report ADRs and SDs?”.

**Responses**	**Group A (n = 669)**	**Group B (n = 146)**	**p-value**
Lack of agreement: (“strongly disagree”, “disagree”, and “don't know” responses)	496 (74.1%)	11 (7.5%)	**0.01**
Agreement: (“agree” and “strongly agree” responses)	173 (25.9%)	135 (92.5%)	
**Response characteristics**	**Statistical values**		
Range	1.0–5.0	1.0–5.0	
Median	3.0	5.0	

Responses to the workplace questions in the second section of the questionnaire show that 10.4% of nurses from Poland think they have enough time to report ADRs and SDs compared with 66.4% of respondents in group B (Chi-squared test p = 0.01). 44.1% of subjects in group A and 91.3% in group B regarded their access to equipment and forms was “adequate” (Fischer test p < 0.001). Subjects in group A (17%) and in group B (90.6%) responded that they are motivated to report problems associated with the safety of pharmacotherapy (Fischer test p < 0.001). The results are presented in Table 3.

**Table 3:** Responses of the Polish (group A) and the American (group B) nurses to the questions concerning the assessment of their workplace in terms of having time at their disposal, unlimited access to equipment, and motivation provided by their employer to report ADRs and SDs.

Question	Answer	Group A n = 669	Group B n = 146	p value
Do you have enough time to report ADRs and SDs?	Lack of agreement (“strongly disagree”, “disagree”, and “don't know” responses)	599 (89.5%)	49 (33.6%)	0.01
	Agreement (“agree” and “strongly agree” responses)	70 (10.5%)	97 (66.4%)	
Do you have adequate access to equipment and forms to report ADRs and SDs?	Lack of agreement (“strongly disagree”, “disagree”, and “don't know” responses)	374 (55.9%)	13 (8.9%)	< 0.001
	Agreement (agree and strongly agree responses)	295 (44.1%)	133 (91.1%)	
Are you motivated in your workplace to report ADRs and SDs?	Lack of agreement (“strongly disagree”, “disagree”, and “don't know” responses)	555 (83.0%)	14 (9.6%)	< 0.001
	Agreement (“agree” and “strongly agree” responses)	114 (17.0%)	132 (90.4%)	

In the third section on attitudes about reporting ADRs and SDs, 15.1% of respondents in group A “strongly agree” and “agree” with having this duty, compared with 91.8% of group B. Conversely, 70.1% in group A “strongly disagree” and “disagree” compared with 4.8% in group B (p < 0.001). It was shown that levels of aspiration to report ADRs and SDs increased in correlation with nurses’ higher education levels (with a correlation coefficient of R = 0.09, p = 0.0232). There was a very weak correlation between nurses’ aspirations to report the adverse effects of medicinal products and their increasing seniority (R = -0.12, p = 0.0014); and similarly with the increasing age of nurses (with a correlation coefficient of R = -0.12, p = 0.0092). The duty to report ADRs and SDs was judged positively by young nurses with low job seniority and high educational level.

To the question “Does the reporting of single cases of ADRs and SDs have any impact on the safety of pharmacotherapy?” 72.4% of group A and 77.4% of group B responded with “strongly agree” and “agree. Conversely, 14.9% of respondents in group A and 9.8% in group B (p < 0.001) answered “strongly disagree” and “disagree”.

Respondents’ answers to the question “Reporting adverse drug reactions is associated with a liability risk for the reporting person?” were mostly negative, with 75.2% in group A and 82.2% in group B indicating “disagree” and “strongly disagree”. Conversely, 9.1% from A group and 7.5% from B group answered with “strongly agree” and “agree”. 15.7% of Polish and 10.3% of American respondents (p < 0.001) answered “I do not know”.

An analysis of nurses’ practice of reporting ADRs revealed that within the period of the previous three years, American nurses had reported ADRs much more frequently (42.5%) than Polish nurses (1%). In group A, 99% of respondents did not report any ADRs in the 3 years prior to the survey, compared with 57.5% in group B. American nurses who had reported ADRs in the previous three years had, during the same period, participated in training sessions concerning the safety of pharmacotherapy (p = 0.0047).

In the previous three years 6.1% of Polish respondents and 19.9% of American respondents had taken no action when a patient reported worrying symptoms after the administration of any given drug. It was revealed that American nurses took action significantly less frequently when a patient reported worrying symptoms which were known and described in the package leaflet (p = 0.0032) or in cases where the reported symptoms were not bothersome for the patient (0.0391). A similar practice was observed when the subjects in group B were certain that any worrying symptoms reported by the patient after receiving a given medicine were not associated with the given drug (p < 0.001) (see Table 4).

**Table 4:** Responses of Polish (group A) and American (group B) nurses to the question: “Did it happen in the previous three years that you took no action when a patient reported worrying symptoms after the administration of any drug?”.

Responses	Group A (n = 669)	Group B (n = 146)	p-value
No	628 (93.9%)	117 (80.1%)	< 0.001
Yes	41 (6.1%)	29 (19.9%)	
• Yes, if they are described in the package leaflet	20 (3.0%)	12 (8.3%)	0.0032
• Yes, if they are not bothersome for the patient	10 (1.5%)	6 (4.1%)	0.0391
• Yes, if I am sure that they are not associated with the given drug	11 (1.6%)	11 (7.5%)	< 0.001

Nurses’ rates of detecting quality defects in medicinal products was 0.4% and 11% (p = 0.0001) in study groups A and B, respectively, for the period of the previous three years of employment. The p values were < 0.001 for the frequency of once a year, 0.0031 for the frequency of twice a year, 0.001 for once in the entire period of employment, < 0.001 for twice in the entire period of employment, and < 0.001 for three times in the entire period of employment.

## 4. Discussion

In our cross-sectional research with Polish and American nurses to evaluate their opinions, attitudes and practices, we found that complacency, time available for reporting (workload), access to forms, availability of equipment needed to send a report, and motivation and lethargy were the main factors associated with nurses’ degree of participation in reporting adverse drug reactions and detecting substandard drugs.

Our analysis of international literature on nurses’ participation in ADR reporting revealed that reporting rates are dependent on individual attitudes, as well as on personal and professional factors and that underreporting of ADRs is associated with specific attitudes of health professionals to ADRs and the reporting system [10]. The literature points to three types of reason. There are those associated with ADR-related knowledge and attitudes, such as complacency, diffidence, indifference, ignorance, and insecurity. Then there are attitudes relating to professional activity, including financial incentives, legal aspects, and whether there is an ambition to publish. Finally, there are excuses for under-reporting, such as lethargy [12].

It is a common belief that drugs are safe. The conviction that all the ADRs of any given drug are known by the time it comes to market and that only safe medications are marketed may lead to errors and dangerous incidents. In fact, drugs that are marketed and authorised for use may not be safe in every clinical case. Lopez-Gonzalez, Herdeiro and Figueiras published a systematic review that revealed complacency (47%) as one of the primary reasons for under-reporting ADRs [3]. The Polish subjects in our study assessed that administered drugs were “somewhat” and “definitely” safe in 71% and 8.2% of cases, respectively. In comparison, the responses from American nurses were 50.7% and 38.4%, respectively. A study performed in Poland by Zięzio (2008), similarly indicated that 57% of nurses consider the pharmacotherapy in their facility to be safe, and 37% as definitely safe [13]. This belief may lead to complacency. Though nurse complacency has negative implications for reporting rates, it is an attitude that can be remedied easily through educational interventions [3].

Low ADR reporting rates are significantly influenced by ignorance and indifference [3]. Lack of knowledge about the functioning of the spontaneous ADR reporting system is ignorance. It has been proven that many professionals believe that the spontaneous ADR reporting programme is exclusively designed to detect severe reactions, yet in reality, all potential types of undesirable effects associated with a given drug are relevant in terms of its safety profile [3]. Our results show that respondents from both groups believe that every single report is important. We did not observe any indifference in this manner.

Most of the participants in our study working in northern Poland, indicated that they are not sufficiently prepared to be able to report ADRs and SDs. On the other hand, the USA participants in our study feel they are adequately prepared to independently report ADRs. It is noteworthy that in our study, only 1.0% of the Polish respondents had reported ADRs compared with 42.5% of the American respondents. Italian studies have revealed that only 6.7% of nurses are reporting ADRs to the Italian Centre for Pharmacological Monitoring [14]. Hanafi et al., (2012) indicated that in their Teheran-based study only 9% of nurses had any experience with reporting ADRs, and that 91% of nurses had never reported adverse reactions [15]. Polish nurses are reporting ADRs at an extremely low rate.

In the three years prior to their participation in our study, only 6.1% of Polish respondents and 19.9% of American respondents took no action when a patient reported worrying symptoms after the administration of a drug. Adverse reactions that are described in the drug’s packaging or leaflet were not reported by 3% of respondents in Poland and 8.3% in the US; a finding that is similar to those in studies conducted in Italy, China and Sweden [14, 16, 17]. 1.5% of respondents in Poland and 4.1% in the US did not report when they noticed that the ADR symptoms were not too bothersome for the patient. 1.6% of Polish and 7.5% of American respondents did not report suspected adverse reactions in cases where they were uncertain whether the reaction was related to the given drug; and this rate is similar to those reported in other studies [18, 19].

In our study, as many as 601 (89.9%) of the Polish subjects had not participated in any training concerning pharmacotherapy safety in the three years preceding their inclusion in the study. Studies concerning the safety of pharmacotherapy and the reporting of adverse reactions demonstrate a need for life-long education of medical personnel. In an Indian study, residents (96%) as well as nurses (91%) suggested that it was their participation in conferences and continued medical education that led to a better understanding of PhV [19]. In the United Arab Emirates, 86.6% of subjects emphasized the need for training in reporting adverse reactions [18]. Training for medical personnel is a key solution and is indispensable for improving the adverse reaction reporting rate. A definitive and successful method for improving reporting would be to include pharmacovigilance into programs of life-long and postgraduate education and to present more detailed guidelines for the spontaneous reporting of ADRs [20].

The risk of being caught up in litigation is one of the factors underlying non-reporting. However, it is interesting that neither the Polish (75.2%) nor the American nurses (82.2%) in our study considered reporting ADRs as an additional liability risk for the person reporting. This absence of legal insecurity was similar to the findings of studies with Portuguese and Indian subjects [19, 21]; and contrasted with studies in Iran where the fear of legal claims was one of the main factors (87%) for not reporting ADRs [22].

It was proven that time spent on activities which do not directly serve health, the availability of the appropriate forms and equipment, and a well-functioning incentive scheme introduced by the employer all play a key role in reporting adverse drug reactions [3]. Our study reveals that as many as 83.9% of Polish nurses declared that they do not have enough time to report ADRs. On the other hand, American nurses perceived their availability at work differently. Most of them (66.4%) declared that they have enough time to report such reactions. Studies from the United Arab Emirates, Portugal and Saudi Arabia revealed that the shortage of time significantly prevented nurses from reporting ADRs [18, 21, 23]. Ekman et al., (2009) found that approximately one third of Swedish nurses (30%) considered they do not have enough time to report adverse reactions [17].

Our study showed there was a substantial technical difference between respondents’ workplaces in Poland and the US. Polish respondents (83%) in our study suggested they had limited access to equipment or reporting forms. On the other hand, most American nurse respondents (91.3%) indicated that they were provided with good access to such tools. The results of a study performed by Muraraiah (2011) in India and other carried out in Nigeria, Saudi Arabia, Italy, Sweden and the United Arab Emirates revealed that the lack of equipment in hospitals prevented their subjects from reporting adverse reactions [14, 17, 18, 23–25]. Our results confirm previous findings that access to forms and equipment needed to send a report play an underlying role in non-reporting in nursing practice.

Most of the Polish nurses included in our study (58.2%) indicated that there is no incentive scheme, or that they are not directly encouraged to report ADRs in their workplace. An additional 24.8% of respondents had “no opinion in this matter” which infers a lack of motivation. It is noteworthy that respondents in the USA report that their employers motivate them to report adverse reactions, as 90.6% of subjects gave a positive response to the question whether they were “definitely” targeted to undertake such actions. Some employers may themselves be motivated by facility reporting requirements.

Results from our study might indicate a kind of lethargy among Polish respondents. In our Polish group, 15.1% of respondents “strongly agree” and “agree” with having the duty to report, compared with 91.8% of respondents in group B. Conversely, those responding with “strongly disagree” and “disagree” were 70.1% in group A compared with 4.8% in group B. The results of studies carried out in the United Arab Emirates show that 40.7% of nurses considered reporting ADRs to be their professional duty, while 31.9% of nurses did not consider it necessary [18]. Over 82% of nurses in a teaching hospital in Tehran did not consider that ADR reporting was a part of their professional duties [20]. In a study in India 75% of nurses consider ADR reporting to be a necessity [19]. Our finding is particularly interesting because, in contrast to the mandatory requirement to report in Poland, ADR reporting by health professionals to the federal government is voluntary in the United States [26–28]. In Poland, reporting ADRs is a legal obligation and yet levels of nurse involvement in ADRs reporting are exceptionally low. This comparison between Polish and American nurses’ attitudes, opinions, and practice in the light of legal obligations compared with voluntary reporting provides a unique set of results for nursing research. It might show that a legislated requirement is not sufficient to encourage satisfactory levels of reporting.

The safety of medicines is related to their quality. Although the possibility of any error is minimized at every stage of the drug manufacturing process, the risk of producing a drug product with a quality defect always remains. Considerable financial outlays and much attention given to quality have led to the situation where quality defects are detected prior to drug dispensing at a rate of up to 99.6% [29]. In clinical practice health care professionals play a key role in identifying and rectifying errors before there is a risk of patients being affected by SDs [29–32]. The World Health Organization indicates that less than 1% of drugs in developed countries and over 10% of drugs in developing countries may be of low quality or counterfeit [33]. Inspection and counterfeit detection procedures are better executed in developed countries, allowing them to undertake adequate actions [32].

According to the Polish Pharmaceutical Inspectorate, medical products available in retail or hospital pharmacies and rural pharmacy shops are safe, yet this assertion must not promote a false sense of security. The results of our own study carried out in northern Poland show that, over the three years prior to our survey, only 0.4% of cases of quality defects were directly reported to the State Pharmaceutical Inspectorate by the nurses we questioned, who made a total of ten such reports. In the group of American nurses, 11% of respondents declared that in the period of the previous three years they had reported such cases which indicates a greater awareness of the problem of quality defects of medicines.

Our study included an assessment of factors relating to the detection and reporting of SDs. Limited studies describe this issue. This make our study unique for nursing practice.

The potential limitations of this work should be noted. Our results could be limited by small sample size in a comparative group. Differences in workload between American and Polish nurses were not considered.

## 5. Conclusions

Our study results indicate that nursing personnel in Poland are insufficiently prepared to ensure drug safety in a conscientious and fully responsible manner. They show that legal regulations are not enough to improve strategies for ADR reporting and SD detection in nursing practice. It is therefore necessary to introduce regular training programs for nurses to improve their knowledge and skills in pharmacotherapy safety. Nurses also need motivational tools to report such incidents to the appropriate authorities.
